# Achieving malaria-free: Egypt's journey to WHO certification and global implications for disease control

**DOI:** 10.1186/s41182-024-00666-5

**Published:** 2024-12-27

**Authors:** Blessing Olawumi Amisu, Olalekan John Okesanya, Mohamed Mustaf Ahmed, Sohaila Mohamed Mohamed Abdelbar, Don Eliseo Lucero-Prisno

**Affiliations:** 1 Department of Medical Laboratory Science, State Hospital Ede, Osun, Nigeria; 2 Faculty of Medicine, Department of Public Health and Maritime Transport, University of Thessaly, Volos, Greece; 3 Faculty of Medicine and Health Sciences, SIMAD University, Mogadishu, Somalia; 4 Kasralainy Medical School, Cairo University, Cairo, Egypt; 5 Department of Global Health and Development, London School of Hygiene and Tropical Medicine, London, UK; 6 Research and Innovation Office, Southern Leyte State University, Leyte, Philippines; 7 Research and Development Office, Biliran Province State University, Biliran, Philippines

**Keywords:** Malaria-free certification, Egypt, WHO, Public health, Disease control, Africa, Vector-borne diseases, Global health strategy

## Abstract

Egypt's recent malaria-free certification by the World Health Organization (WHO) marks a significant achievement in public health, underscoring the effectiveness of sustained national efforts in disease eradication. This milestone, achieved after nearly a century of strategic intervention, highlights the importance of integrated public health programmes and cross-sector collaboration. Egypt's journey involved early initiatives to reduce human–mosquito contact, the establishment of malaria control stations, and comprehensive outbreak management strategies. This country's success serves as an exemplar for other African nations, emphasizing the need for adaptable, community-focused approaches to disease control. Despite challenges such as drug-resistant malaria strains and pesticide-resistant mosquitoes, Egypt's experience demonstrates the potential for successful malaria elimination through coordinated efforts and innovative solutions. This accomplishment contributes to regional health improvements, and provides valuable insights into global malaria eradication strategies.

Dear Editor,

A significant public health milestone for a nation of over 100 million people was reached when the World Health Organization (WHO) formally declared Egypt malaria-free [1]. In the WHO Eastern Mediterranean Region, Egypt has received malaria-free certification since 2010, third after Morocco and the United Arab Emirates. This achievement culminated in nearly a century of concerted efforts by the Egyptian government to eradicate malaria that has existed there since ancient times. However, it is important to clarify that elimination refers to the cessation of malaria transmission in a specific region, as opposed to eradication, which denotes the global clearance of a pathogen, a feat achieved only with smallpox to date. This accreditation highlights the efficacy of public health programs, the importance of tailoring interventions to regional malaria dynamics, and continuing investments in disease prevention and control. Additional insights into the actions and policies adopted are crucial for providing a roadmap for malaria endemic nations. Egypt’s malaria transmission patterns are more similar to those of other certified countries in this region than those observed in Sub-Saharan Africa (SSA) [1]. Forty-four countries and one territory worldwide achieved this milestone. The importance of this accomplishment cannot be overemphasized, particularly in light of the highly varying prevalence of malaria transmission challenges between regions, such as the Eastern Mediterranean and SSA [2].

The strategies that led to Egypt’s malaria-free status can be divided into three key phases: initial efforts to reduce human–mosquito contact, large-scale outbreak management, and sustained elimination measures. The first steps to lessen human–mosquito contact occurred in Egypt in the 1920s when the government forbade growing rice and other crops close to residential areas [3]. The restriction of rice planting within 5 km of residences significantly reduced mosquito breeding sites but posed challenges for subsistence farmers. Alternatives such as relocating rice cultivation to less densely populated areas were explored to balance agricultural productivity and vector control [3]. Egypt created its first malaria control station in 1930, concentrating on diagnosis, treatment, and monitoring, and declared malaria as an illness that needed to be reported. This effort is especially important because a large section of the population lives along the banks of the Nile River, where up to 40% of the population may contract malaria [1]. The number of malaria cases in Egypt increased to over three million by 1942 due to several causes, including World War II-related population dislocation, disruptions in medical supplies and services, and the invasion of *Anopheles arabiensis*. Egypt responded to this crisis with a strong control strategy, which eventually resulted in successful management of malaria. This strategy included the creation of 16 treatment divisions and hiring almost 4000 health personnel [1].

Egypt's Ministry of Health and Population concentrated on preventing the reestablishment of local malaria transmission after the disease was successfully controlled by 2001. One pivotal strategy implemented was the rapid containment of outbreaks, such as the 2014 malaria outbreak in the Aswan Governorate, through public information campaigns, early case detection, timely treatment, and targeted vector control interventions. These actions underscore the importance of integrating robust surveillance systems with community-level responses [1]. Over the last ten years, Egypt has made significant progress in improving its citizens' access to healthcare services. For instance, 95% of Egyptians live 5 km or less from a primary healthcare center, which is a noteworthy sign of this achievement. Additionally, all citizens, including undocumented migrants from Sudan, are eligible for free malaria diagnosis and treatment by the government [4]. Egypt has successfully combined the management of other vector-borne diseases with its attempts to control malaria. The Ministry of Agriculture, Ministry of Environment, and Ministry of Water Resources and Irrigation all had representatives from the High Committee for Integrated Vector Management, which was formed in 2016. The committee adopted an integrated vector management approach, securing funding and harmonizing efforts across various ministries to address disease prevention comprehensively. These measures highlight Egypt's commitment to sustained investment in disease control and the importance of intersectoral collaboration [4]. The impact of malaria interventions in Egypt, illustrated through a trend analysis, showed that malaria cases in Egypt dropped from over 3 million in the 1940s to near zero by 2001. This decline demonstrates the cumulative success of integrated control strategies, including surveillance, treatment, and public health outreach [1]. The fact that other African countries, including those in the Southern African Development Community (SADC) region, are making progress in their efforts to eradicate malaria, largely via the Elimination 8 initiative, suggests that this goal may be accomplished. However, drug-resistant forms of malaria and pesticide-resistant mosquito populations remain problematic [5]. Utilizing the current infrastructure and resources is essential for maintaining efforts to treat malaria and other infectious illnesses, as the WHO has set an ambitious goal of reducing malaria cases and deaths by 90% by 2030 [6].

In light of Egypt’s achievements, it is crucial to recognize that a single approach is unlikely to be successful across a variety of settings because of the complexity of malaria transmission [7]. A sustainable strategy must be flexible, long-term, and context specific, focusing on community involvement, integration with current health systems, and responsive surveillance frameworks. It is equally important to address Egypt’s mosquito ecology and contextualize lessons for other African nations, where climatic and vectorial differences may limit the direct applicability of Egypt’s strategies. Specific recommendations for countries with similar epidemiological and ecological profiles would help ensure the targeted application of Egypt’s experience. For SSA, integrating these strategies with solutions for high transmission settings, such as vector-targeted interventions and responsive surveillance, is critical. Achieving this goal in SSA requires addressing unique challenges and tailoring interventions in the local epidemiological context. The Lancet Commission for Malaria Eradication and the WHO Strategic Advisory Group emphasize that while elimination is feasible in certain regions, achieving eradication will demand innovative, multi-disciplinary approaches and unprecedented levels of global cooperation [8]. African nations should adopt a multilayered proposition marked by capable leadership, flexible surveillance systems, demand-driven scientific research, capacity building, wide-ranging cooperation, gap analysis, strong healthcare systems, and community engagement [9].

The WHO's global malaria policy calls for nations where malaria is endemic to expedite efforts to eradicate the disease. It is necessary to implement population-wide techniques to decrease malaria transmission in places where the disease is widespread among the people in a certain geographic area (such as a district or village) [6]. Mass relapse prevention, medication delivery, testing, and therapy are examples of these tactics. It is typically not advisable to use mass tactics in post-elimination settings, unless local malaria transmission resumes. For higher-risk groups, focused therapies such as targeted drug administration (TDA) are advised, whereas reactive strategies, such as reactive case detection and treatment (RACDT) are crucial for lowering transmission clusters [6].

A significant change in the region's attempts to prevent and control malaria has been brought about by the recent introduction of malaria vaccination throughout Africa. This implementation is a significant step forward, especially for the most susceptible children under the age of five years. To guarantee the long-term advancement and fair distribution of these vaccines, Africa must address financial obstacles and improve the cooperation and coordination required for effective execution of mass vaccination campaigns [10, 11]. Egypt's success in eliminating malaria provides a powerful example of regionally tailored strategies. By drawing lessons from Egypt’s extraordinary progress in the WHO Eastern Mediterranean Region, African nations can craft innovative, context-specific approaches to combat malaria and pave the way for a malaria-free future. This vision may become a shared reality if we collaborate with each other.

## Data Availability

No datasets were generated or analysed during the current study.
